# Nuclear shape, architecture and orientation features from H&E images are able to predict recurrence in node-negative gastric adenocarcinoma

**DOI:** 10.1186/s12967-019-1839-x

**Published:** 2019-03-18

**Authors:** Meng-Yao Ji, Lei Yuan, Xiao-Da Jiang, Zhi Zeng, Na Zhan, Ping-Xiao Huang, Cheng Lu, Wei-Guo Dong

**Affiliations:** 1 Department of Gastroenterology, Wuhan University Renmin Hospital, Wuhan, Hubei China; 2 Department of Information Center, Wuhan University Renmin Hospital, Wuhan, Hubei China; 3 Department of Pathology, Wuhan University Renmin Hospital, Wuhan, Hubei China; 4 Department of Gastroenterology, The Central Hospital of Wuhan, Tongji Medical College, Huazhong University of Science and Technology, Wuhan, 430060 China; 5 College of Computer Science, Shaanxi Normal University, Xi’an, Shaanxi China

**Keywords:** Digital H&E images, Predication, Negative-node gastric adenocarcinoma, Quantitative histomorphometric

## Abstract

**Background:**

Identifying intestinal node-negative gastric adenocarcinoma (INGA) patients with high risk of recurrence could help perceive benefit of adjuvant therapy for INGA patients following surgical resection. This study evaluated whether the computer-extracted image features of nuclear shapes, texture, orientation, and tumor architecture on digital images of hematoxylin and eosin stained tissue, could help to predict recurrence in INGA patients.

**Methods:**

A tissue microarrays cohort of 160 retrospectively INGA cases were digitally scanned, and randomly selected as training cohort (D1 = 60), validation cohort (D2 = 100 and D3 = 100, D2 and D3 are different tumor TMA spots from the same patient), accompanied with immunohistochemistry data cohort (D3′ = 100, a duplicate cohort of D3) and negative controls data cohort (D5 = 100, normal adjacent tissues). After nuclear segmentation by watershed-based method, 189 local nuclear features were captured on each TMA core and the top 5 features were selected by Wilcoxon rank sum test within D1. A morphometric-based image classifier (NGAHIC) was composed across the discriminative features and predicted the recurrence in INGA on D2. The intra-tumor heterogeneity was assessed on D3. Manual nuclear atypia grading was conducted on D1 and D2 by two pathologists. The expression of HER2 and Ki67 were detected by immunohistochemistry on D3 and D3′, respectively. The association between manual grading and INGA outcome was analysis.

**Results:**

Independent validation results showed the NGAHIC achieved an AUC of 0.76 for recurrence prediction. NGAHIC-positive patients had poorer overall survival (P = 0.017) by univariate survival analysis. Multivariate survival analysis, controlling for T-stage, histology stage, invasion depth, demonstrated NGAHIC-positive was a reproducible prognostic factor for poorer disease-specific survival (HR = 17.24, 95% CI 3.93–75.60, P < 0.001). In contrast, human grading was only prognostic for one reader on D2. Moreover, significant correlations were observed between NGAHIC-positive patients and positivity of HER2 and Ki67 labeling index.

**Conclusions:**

The NGAHIC could provide precision oncology, personalized cancer management.

**Electronic supplementary material:**

The online version of this article (10.1186/s12967-019-1839-x) contains supplementary material, which is available to authorized users.

## Background

The gastric cancer (GC) is a common gastrointestinal tumor with high mortality and the second leading cause of death in China [1]. For these cases, 80% are gastric adenocarcinoma (GA). Nodal metastases are a well-known prognostic factor after radical treatment of gastric cancer. Because intestinal node-negative gastric adenocarcinoma (INGA) patients have a good prognosis, it remains controversial whether adjuvant chemotherapy is needed for INGA patients after surgery. There is controversy surrounding the benefit of adjuvant therapy for patients with resected stage IB, especially pT2N0. The National Comprehensive Cancer Network (NCCN) guidelines suggest for some high-risk cases (pT2N0 with a high histologic grade or the presence of lymph vascular or perineural invasion), the decision to pursue adjuvant therapy should be personalized. Observation is appropriate for patients with resected T2N0 stage IB GC as long as they have undergone adequate lymph node dissection. But guidelines from the European Society for Medical oncology (ESMO) suggest adjuvant therapy for all patients with resected stage IB disease, including those with pT2N0 tumors. NCCN and ESMO recommended adjuvant therapy for all patients with pT3-4N0. Observation without adjuvant therapy for patients with T1N0 who have uninvolved section margins. For patients with early-stage gastric cancer, the risk of lymph node metastasis is low (2–28 percent for T1, 20 percent for T2). While chemotherapy has many side effects, such as loss of hair, myelosuppression, damage to liver and kidney, and additional extensive medical burden, it is critical to distinguish recurrence in INGA patients perceived benefit of adjuvant chemoradiation after an R0 resection.

Although there were many predicative factors for recurrence and could be useful to stratify node-negative gastric cancer patients for adjuvant treatment and tailored follow-up, including lymphatic embolization and perineural infiltration, p53 and Ki67 and greater lymph node retrieval. But these assays are tissue destructive and expensive. Pathologic staging (e.g. nuclear atypia grade) is critical in directing optimal treatment for INGA patients. Unfortunately, pathological analysis is a tedious process and suffered from intra/inter-reader variability.

Computer-aided image analysis has great potential to conquer inconsistencies in virtue of subjective interpretation [2–5]. Quantitative histomorphometry (QH) used computer-aided image analysis to decrypt sub-visual differences of tumor morphology in digital pathology images. With the advancement of computer-aided image technologies, a number of quantitative morphology information was extracted and has been approved to be prognostic, such as tumor nests fractal dimension and stromal morphologic features [6, 7].

Recent reports have shown that nuclear architecture was useful in cancer grading and predicting patient outcomes [2, 5, 6, 8–13]. Genetic instability could be displayed by diversify of nuclear shape and texture, playing important role in metastasis and proliferation that result in cancer recurrence potentially [11, 12]. Quantitative histomorphometry of nucleus architecture was utilized to predict disease recurrence in early-stage non-small cell lung cancer [14], biochemical recurrence [11] for prostate cancers [11, 15] and so on [6, 10, 12].

**Fig. 1:**
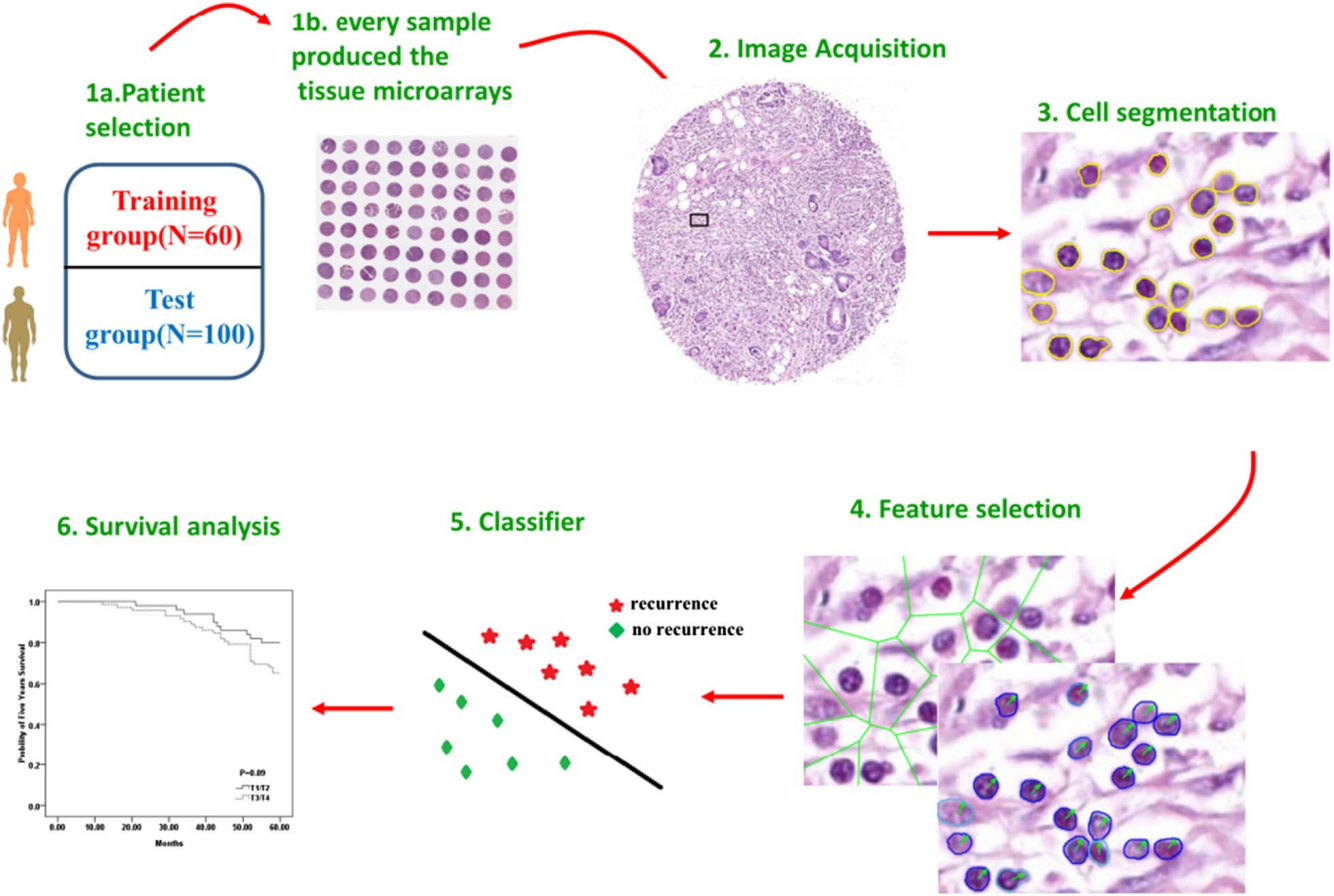
Illustrations of work flow for this study

In this study, we constructed a quantitative histomorphometry based model to distinguish INGA patients who suffered from recurrence versus those did not using a cohort of 60 TMA images. We then validate the model in another validation cohort of 100 TMA images. The work flow of this study was illustrated in Fig. 1. With the help of image classifier, we are looking forward to identify patients who have high risk of recurrence and who might thus receive measurable benefit adjuvant chemotherapy after curative resection.

## Materials and methods

### Study population

With the approval of the ethical board of Renmin Hospital of Wuhan University (Wuhan, Hubei, China) and abided with the Declaration of Helsinki, 1782 candidate patients with GA were collected from Department of Pathology, Renmin Hospital of Wuhan University archives from 2000 to 2012 retrospectively and consecutively. Two pathologists (Z.Z and N.Z) were then assigned to identify the patients with node-negative gastric adenocarcinoma. Subsequently, the corresponding donor blocks and their H&E stained slides were obtained followed by selecting preferred blocks and marking areas of interest for core punching. Three to six cores of 2 mm in diameter was punched from central tumor/leading edge of the donor block ROI using a thin-wall stainless steel tube and transferred onto the recipient blocks to construct the arrays. The digital H&E images were captured under the Aperio Scan Scope XT Slide Scanner at 40× magnification with a resolution of 0.25 μm per pixel. One of the most representative tumor cores were selected by Z.Z and Z.N for use. The enrolled spots were randomly divided into training cohort (D1 = 60 [37.5%]) and test cohort (D2 = 100 [62.5%]), respectively.

Additionally, three 2 mm punches were removed from different tumor region of D2 and assessed by pathologists for prefer spots after digitally scanning under slide scanner, as well as two 2 mm punches from normal adjunct tissue as negative control. Finally, a third dataset, named D3 (n = 100), was also recruited in this study, containing tissue cores corresponding to the same patients in D2 but extracted from different regions of the tumor. D3 was employed to validate the image classifier to cope with tumor heterogeneity and immunohistochemical staining for HER2. A fourth dataset, named D3′, was duplicate cohort of D3, using for immunohistochemical assessment of Ki67. A fifth dataset, named D5 (n = 100), was obtained from the adjacent normal tissues of D2 as negative controls. D1 contains D+ (recurrence) patients and D− (non-recurrence) patients. In contrast, for D2, D3 and D4, only the digital H&E images were used to predict the recurrence status without any pre-knowledge of the patients.

In this paper, the INGA samples dataset was selected based off the after mentioned inclusion/exclusion criteria. Inclusion criteria comprised of: (1) pathological diagnosis of gastric adenocarcinoma; (2) according to the standard of gastric cancer TNM staging by Union for International Cancer Control: gastric adenocarcinoma TMN stage was limited to pT1–T4N0M0 before postoperative pathology; (3) After radical surgery, more than 16 lymph nodes were selected for biopsy; (4) recurrence or metastasis was confirmed by CT or MRI images, endoscopy, and pathology; (5) complete clinic pathological data (through telephone, data collected by clinicians or database of electronic medical records and all patients were followed up for 5 years). Accompanying exclusion criteria contained: (1) patients with other primary malignant tumors; (2) patients who underwent chemotherapy or immunotherapy before surgery; (3) palliative surgery; (4) other diseases or accidental deaths; (5) residual gastric cancer; (6) lost visits or incomplete data; (7) death within 1 month after operation. The period of no recurrence and metastasis was limited from the time after the surgery to the diagnosis of recurrence or the time of final follow-up. The period of recurrence or metastasis was limited from the time of diagnosis, recurrence or metastasis to death or final follow-up time; the overall survival time was from surgery to death or the last follow-up time. The deadline for follow-up was on December 31st, 2017.

### Image analysis

#### Nuclear segmentation

**Fig. 2:**
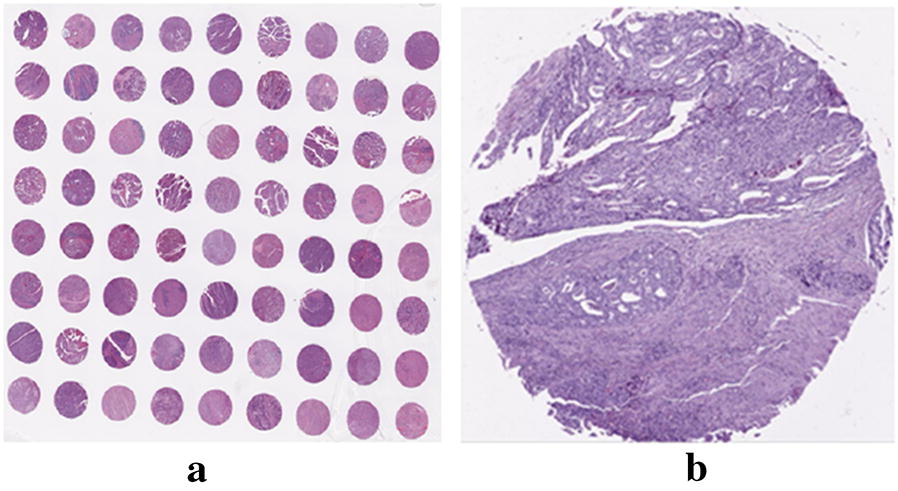
It was shown digital pathological H&E image of INGA tissue. **a** Digital pathological H&E image of INGA tissue microarray. **b** Digital pathological H&E image of one INGA tissue microarray spot

Each individual nucleus, including cancer tissue and tumor stroma, was detected and segmented by Watershed-based nuclear segmentation method [16] at 40× magnification (0.25 μm/pixel resolution) automatically after color deconvolution for isolating the stain. This resulted in a RGB color digital image for each TMA spot, the similar one was shown in Fig. 2.

#### Feature extraction

Three different types of quantitative histomorphometric cellular features, covering local architectural features, shape/texture features, local Cell Orientation Graphs features, were extracted from local cluster regions [6, 8] in this study. Of these features, 20 nuclear architectural features, with 12 features from Voronoi Diagram and 8 features from Delaunay Graph, were extracted aimed to capture the nuclear architectural disorder in local regions indicating more aggressive tumor behaviors. 39 nuclear orientation disorder features related to nuclear orientation disorder were derived from Cell Orientation Graphs [10]. 100 shape features and 30 nuclear texture features, comprised of invariant moment, Fourier descriptors of boundary, area, length/width ratios, smoothness, perimeter ration, and area ration and so on, were extracted as described in ref. [17] aiming to capture the disorder linked to shape/texture disorder in local cluster regions. Finally, a total of 189 features were yielded for each TMA core (Table 1) in our study. A comprehensive list of all quantitative features was shown in Additional file 1: Table S1.

**Table 1:** Summary of histomorphometric features extracted from TMA

Feature type	No.	Description
Nuclear shape	100	Area ratio, distance ratio, SD of distance, distance ratio, perimeter ratio, variance of distance, fractal dimension, smoothness, invariant moment 1–7, Fourier descriptor 1–10: min/max, mean, SD, median
Nuclear texture	30	Contrast, energy, entropy, inverse variance, invariant moment: mean, SD from each channel
Nuclear orientation map	39	Contrast energy, contrast inverse moment, contrast average, contrast variance, contrast entropy, intensity average, intensity variance, intensity entropy, entropy, energy, correlation, information measure 1, information measure 2: mean, SD, range
VD	12	Perimeter, chord, area: SD, min/max, disorder, average
DT	8	Side length, triangle area: min/max, mean, standard deviation, median, disorder
In total	189	

*SD* standard deviation, *VD* Voronoi diagram, *DT* Delaunay triangulation

#### Feature selection

Three different feature selection schemes, including the minimum redundancy maximum relevance (MRMR), Wilcoxon rank sum test (WRST), and random forest (RF), were employed to identify the most outstanding pathological morphometric features in the training groups. A randomized threefold cross-validation scheme along with 100 iterations, combing with each feature selection method, was used to guarantee the robustness of the preferred the features. These approaches resulted in a total of three accompanying feature bins for disguising the recurrences and non-recurrence cases within the training group, respectively. In this paper we limited the number of candidate features to 5 aimed to avoid curse of dimensionality or over fitting challenges using box and whisker plots. Each feature bin consisted of 5 most distinguished features accordingly, and these features were considered as a prerequisite for inclusion in subsequent classifier construction procedure.

#### Classifier construction

Four different machine learning schemes, comprising of analysis of linear discriminant (ALD), analysis of quadratic discriminant (AQD), machine of support vector (MSV), and random forest (RF), in conjunction with the 3 feature bins, resulting in 12 different machine learning combination modes, were applied to construct the candidate histopathological image classifiers for INGA patients (denoted as NGAHICs) within training group. This full join between machine learning scheme and feature bin gave rise to 12 different optional NGAHICs successfully. Subsequently, the optimal histological image classifier (NGAHIC) was settled down across each candidate classifier productivity (AUC, area under the receiver operating characteristic curve) within the training group. Of note, each binary histological image classifier yield a predictive probability value for distinguishing recurrence or non-recurrence case. In this study, the recurrence level was set at 0.5 empirically, namely the NGAHIC predictive probability value (> 0.5) on each core was considered to be recurrence case. All the binary classifiers predictive outcomes were compared with the ground truth label for classifier performance evaluation.

### Nuclear atypia grade by human readers

Since the histomorphometric features we investigate related to nuclear atypia, key predictors of prognosis in various cancers [2, 5, 6, 8–15, 18–20]. However, only the modest agreements were achieved among experienced readers [13]. We designed the comparative strategies, aiming to illustrate the pathologist’s inter-reader variability in INGA recurrence prediction and compare the prognostic performance of image classifier against subjective manual nuclear atypia grading. The nuclear atypia grade estimation was conducted by two expert pathologists (Z.Z and N.Z) via visual evaluation of the H&E images on training set and test set. Both human readers were blinded to the ground truth information of the 160 cases. Each pathologist was asked to assign a score between 0 and 2 for each digital image in-house. The nuclear atypia grade was defined as 0, 1 referring to low nuclear atypia grade and 2 referring to high nuclear atypia grade, based off the previous work by Nakashima [13].

### Immunohistochemistry

All immunohistochemical stains were performed by the following order: deparaffinizing → antigen retrieval → blocking → primary antibody → washing → blocking → biotinylated secondary antibody → washing → blocking → washing → mounting and observation as our previous work [21]. Each core in D3′ and D3 was immunostained using monoclonal antibody against Ki67 (clone MIB-1; 1:200; Dako, Glostrup, Denmark); polyclonal antibody against HER2/neu (1:200; Dako, Glostrup, Denmark), respectively. The HER2 staining results were assigned a score as IHC 0, IHC 1+, IHC 2+ with FISH (fluorescence in situ hybridization) negative referring negative, IHC 2+ with FISH positive and IHC 3+ indicating positive according to the criteria recommended by Min [22]. Ki67 labeling index was scored by the percentage of nuclei-stained cells observing in 5 randomly selected areas of the section with 400× high-power fields; 200 tumor cells were counted in each area. The Ki67 labeling index was determined as positive (≥ 14% reactive tumor cells) and negative (< 14% reactive tumor cells) as described by Goldhirsch [23].

### Survival analysis

Two-sided Fisher’s test was used to analyze the correlations among the data of machine classifier, clinical documents, and pathologic features. Five years survival probabilities were evaluated by the Kaplan–Meier method and log-rank tests were performed to detect recurrent differences. Cox regression model was employed to detect the independently predicted survival of probabilities of variable factors after checking clinical data and pathologic features. The average expression rates of HER2 and Ki67 between NGAHIC-positive and NGAHIC-negative were evaluated using Chi square test. All tests were repeated for three times, hazard ratios, associated 95% confidence intervals, and P values were reported, with the significance level set at 0.05.

## Results

### Baseline characteristics of the study population

One hundred and sixty patients were finally enrolled in the principal cohort. The clinical and pathological features were shown in Table 2. Of the 160 cases of INGA, most of the patients (122/160 [76.3%]) were married and the media age was 62 years. About 60% (95/160) were men, with 41.7% (n = 25) vs. 60% (n = 60) in D1 and D2, respectively. 89 patients (43.3%) were in T1/T2, whereas 71 (44.4%) had advanced disease (T3/T4). 99 of the 160 patients differentiated well vs. 61 cases differentiated poorly. Of those well-differentiated category, 38 (38/60 [63.3%]) were in D1 and 61 (61/100 [61%]) in D2, separately. Approximate 28% (n = 45) NGA patients were treated with postoperative chemotherapy and more than 65% (n = 103) patients’ tumor size < 5 cm. At the endpoint of the follow-up, 36 patients (36/160 [22.5%]) suffered disease recurrence and 40 patients (40/160 [25%]) dead from related cause.

**Table 2:** Clinical pathological feature of the selected patients

Variable	Sub variables	Total (%)	D1 (%)	D2/D3 (%)
Number of patients		160	60	100
Age		62.1 ± 9.0	63.9 ± 5.7	59.1 ± 10.1
Sex	Male	95 (59.4)	25 (41.7)	60 (60.0)
	Female	65 (40.6)	35 (58.3)	40 (40.0)
Patient status	Alive	120 (75.0)	43 (71.7)	77 (77.0)
	Dead	40 (25.0)	17 (28.3)	23 (23.0)
Recurrence	Yes	36 (22.5)	15 (25.0)	21 (21.0)
	No	124 (77.5)	45 (75.0)	79 (79.0)
Tumor diameter (cm)	< 5	103 (65.6)	40 (66.7)	63 (63.0)
	≥ 5	57 (35.6)	20 (33.3)	37 (37.0)
Invasion depth	Out	92 (57.5)	35 (58.3)	57 (57.0)
	In	68 (42.5)	25 (41.7)	43 (43.0)
T stage	T1/T2	89 (55.6)	32 (53.3)	57 (57.0)
	T3/T4	71 (44.4)	28 (46.7)	43 (43.0)
Histology grade	W/M	99 (61.9)	38 (63.3)	61 (61.0)
	Poorly	61 (38.1)	22 (36.7)	39 (39.0)
Postoperative-chemotherapy	Yes	45 (28.1)	17 (8.3)	28 (28.0)
	No	115 (71.9)	43 (71.7)	72 (72.0)
Manual nuclear atypia grading	Low	102 (75.0)	37 (61.7)	65 (65.0)
	High	58 (25.0)	23 (38.3)	35 (35.0)

*Out* out of serosa, *in* invasion of serosa, *W/M* well and moderate-differentiated, *poorly* poorly-differentiated

### Discriminative features

The top 5 discriminative morphologic features identified within the training cohort were range of intensity entropy, range of intensity energy, standard deviation (SD) of perimeter ration, SD of intensify average, and disorder of perimeter, respectively. Notable, the nuclear orientation related morphometric features, (range of intensity entropy, range of intensity energy and SD of intensify average), predominated the discriminated features (3 out of 5). Additional file 2: Table S2 referred a more comprehensive discriminated feature list.

**Fig. 3:**
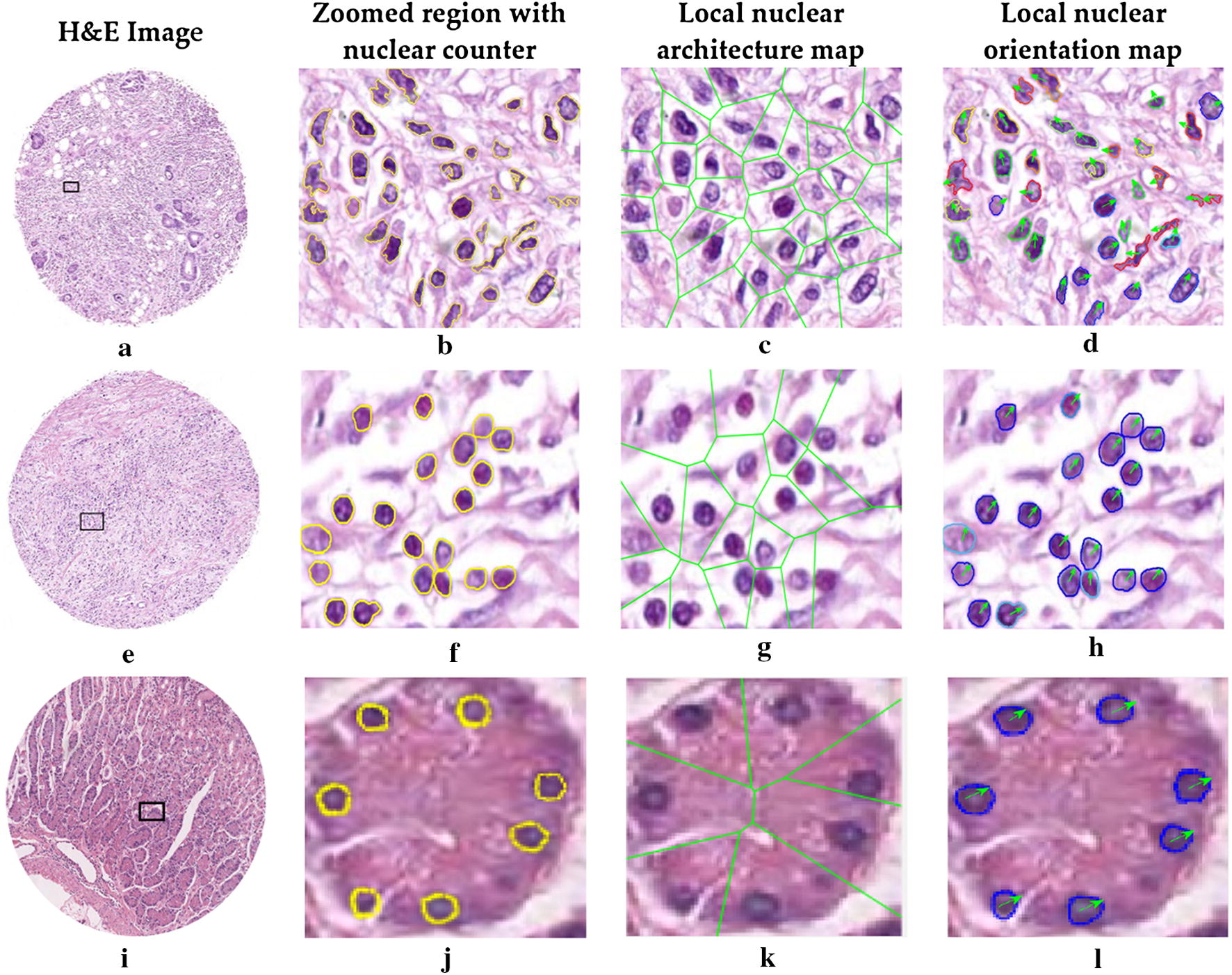
Analysis of digital pathological H&E image of NGA. H&E image from a patient with recurrence (**a**), without recurrence (**e**) and negative controls (**i**). The zoomed region with nuclear counters (**b**, **f**, **j**), nuclear shape, local nuclear architecture maps (**c**, **g**, **k**) and corresponding nuclear orientation maps (**d**, **h**, **l**) were extracted from **b**, **f** and **j**. In **d**, **h** and **l**, the arrows and different colors nuclear contours represent different nuclear orientations. The nuclear architecture feature map appeared to sparser and the nuclear shape and orientation tended to more uniform in local cluster regions (shown in **f**–**h**) for non-recurrence patient, compared with that of recurrence patient (shown in **b**–**d**)

Intuitively, the higher value of the feature was observed, indicating the more distorted of nuclear in local cluster region (Fig. 3). The original H&E digital images (Fig. 3a, e), with accompanying nuclear segmentation contour, nuclear architecture feature map and nuclear orientation feature maps in zoomed region, were shown in Fig. 3, representing recurrence and non-recurrence NGA groups from the first column to the fourth column. For recurrence cases, the nuclear appearance (Fig. 3b, f) showed a bigger variation comparing with the non-recurrences ones. In contrast, the checkerboard architecture feature map appeared to sparser (Fig. 3g) and the nuclear orientation tended to more uniform (Fig. 3h) in local cluster regions in TMA. Comparatively, the nuclear appearance, architecture and nuclear orientation seem to more regular and uniform for the negative controls (Fig. 3j–l).

### Classifier performance

Twelve different machine learning combination modes, resulted from full join between 4 machine learning algorithms and 3 feature selection methods, were conduct on the training group and the corresponding performance results were summarized in Table 3. It is notable that the combination of SVM and WRST yielded the best AUC as well as accuracy, specificity and sensitivity (AUC = 0.87, accuracy = 0.89, specificity = 0.88 and sensitivity = 0.78) in distinguishing D+ and D− within training group. Therefore, this combination scheme (SVM combined with WRST) was settled down as the optimal histological image classifier for predicting NGA recurrence (NGAHIC). In the validation set, the NGAHIC (SVM combined with WRST) yielded an AUC = 0.76, accuracy = 0.72, with corresponding specificity = 0.74 and sensitivity = 0.68 (Table 3).

**Table 3:** Evaluation of different combinations for feature selection and classifier validation on training set and test set

Dataset	Classifier	Feature selection	AUC	Accuracy	Specificity	Sensitivity
D1	ALD	WRST	0.77 ± 0.08	0.81 ± 0.08	0.82 ± 0.05	0.67 ± 0.03
		MRMR	0.67 ± 0.05	0.80 ± 0.04	0.84 ± 0.02	0.69 ± 0.09
		RF	0.76 ± 0.03	0.79 ± 0.06	0.85 ± 0.02	0.62 ± 0.05
	AQD	WRST	0.81 ± 0.02	0.74 ± 0.09	0.80 ± 0.03	0.71 ± 0.08
		MRMR	0.79 ± 0.06	0.77 ± 0.01	0.82 ± 0.01	0.73 ± 0.01
		RF	0.72 ± 0.06	0.87 ± 0.02	0.86 ± 0.03	0.75 ± 0.03
	RF	WRST	0.83 ± 0.03	0.79 ± 0.06	0.82 ± 0.06	0.72 ± 0.04
		MRMR	0.81 ± 0.06	0.76 ± 0.04	0.80 ± 0.08	0.70 ± 0.06
		RF	0.80 ± 0.05	0.73 ± 0.08	0.79 ± 0.06	0.69 ± 0.08
	SVM	WRST	*0.87 ± 0.03*	*0.89 ± 0.02*	*0.88 ± 0.01*	*0.78 ± 0.08*
		MRMR	0.84 ± 0.02	0.88 ± 0.01	0.84 ± 0.02	0.72 ± 0.04
		RF	0.81 ± 0.05	0.84 ± 0.04	0.82 ± 0.02	0.73 ± 0.07
D2	SVM	WRST	0.76	0.72	0.74	0.68

Evaluation values in italic indicate the best machine learning combination

*MRMR* minimum redundancy maximum relevance, *RF* random forest, *WRST* Wilcoxon rank sum test, *LDA* analysis of linear discriminant, *AQD* analysis of quadratic discriminant, *SVM* machine of support vector, *AUC* area under receiver operating curve

### Comparison of human-based nuclear atypia grade and image classifier for predicting recurrence in NGA

**Fig. 4:**
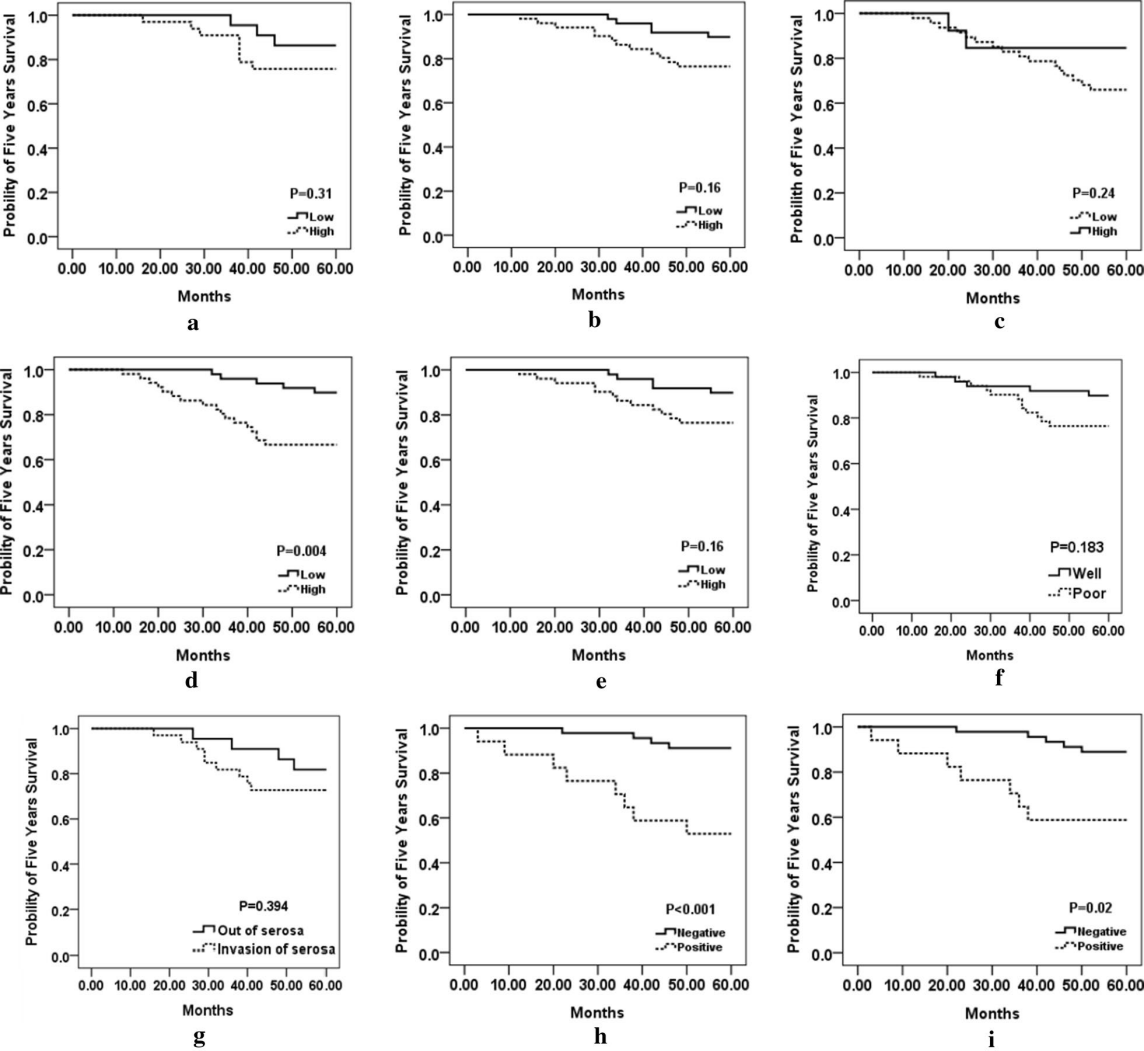
Prognostic prediction results for human readers, NGAHIC, T stage and histology grade. **a**, **b** Kaplan–Meier survival curves for reader 1 on D1 and D2. **c**, **d** Kaplan–Meier survival curves for reader 2 on D1 and D2. **e**–**h** Kaplan–Meier survival curves for T stage, histology stage, NGAHIC and invasion depth on D1, respectively. **i** Kaplan–Meier survival curves for NGAHIC on D3

The Kaplan–Meier curves represented the survival results for both human readers (Z.Z and N.Z) on D1 and D2, respectively (Fig. 4a–d). For reader 1, the estimation of nuclear atypia grade was not significantly correlated with survival outcome for D1 (P = 0.31) nor D2 (P = 0.16). Whereas, for reader 2, there was a statistical significant negative correlation with human-based estimation of nuclear atypia grade and disease outcome for D2 (P = 0.004), but not for D1 (P = 0.24), conversely.

### Correlation of immunohistochemical data and image classifier

HER2 staining was observed in the cytoplasmic membrane of the cancer cells in 16 cases (IHC 0: 36 cases, IHC 1+: 43 cases, IHC 2+ with FISH negative: 5 cases, IHC 2+ with FISH positive: 3 cases and IHC 3+: 13 cases), with the positive rate 81.3% vs. 3.6% in NGAHIC-positive and NGAHIC-negative, respectively. The Ki67 labeling index positive rate was much higher (75.0%) in NGAHIC-positive patients, whereas the Ki67 positive rate was relatively lower (2.4%) in NGAHIC-negative cases. There was statistically significant difference between NGAHIC-positive vs. NGAHIC-negative with positive expression of HER2 (P < 0.001) and Ki67 labeling index (P < 0.001), respectively. More details could be found in Additional file 3: Table S3.

### Survival analysis

All the patients were followed up for 5 years (median survival time was about 38 months). Table 4 showed that the result calculated by univariate log-rank survival analysis for the clinical-pathologic features of the test group. It clearly depicted that the classifier negative patients, got better prognosis compared with classifier positive patients (P = 0.017). Figure 4 showed that prognostic prediction results for human readers, NGAHIC, T stage and histology grade by Kaplan–Meier survival curves. Table 5 demonstrated the results calculated by multivariate survival analysis for the major clinical pathologic features and image classifier. The data showed that there was a strong correlation between the result of NGAHIC and prognosis (HR = 17.24, 95% confidence interval = 3.93–75.60, P < 0.001), indicating NGAHIC was a negative predictive factor for INGA patients independently.

**Table 4:** Univariate log-rank analysis conducted on D2

Variable	HR (95% CI)	P value
Age (< 60 vs. ≥ 60)	0.66 (0.13–3.35)	0.621
T-stage (T1/T2 vs. T3/T4)	2.18 (1.10–4.31)	*0.024*
Histology (W/M vs. poorly)	3.66 (1.12–11.98)	*0.032*
Chemotherapy (yes vs. no)	3.89 (0.92–16.47)	0.065
Invasion depth (out vs. in)	1.87 (1.03–3.39)	*0.039*
Tumor diameter (< 5 cm vs. ≥ 5 cm)	2.15 (0.88–5.22)	0.091
Manual nuclear atypia grading (low vs. high)	3.08 (0.90–10.49)	0.072
NGAHIC (positive vs. negative)	4.14 (1.28–13.29)	*0.017*

*CI* confidence interval, *HR* hazard ratio; *W/M*: well and moderate-differentiated, *poorly*: poorly differentiated, *out*: out of serosa, *in*: invasion of serosa

P value in italic is statistically significant, P < 0.05

**Table 5:** Multivariate survival analysis conducted on D2

Variable	P value	HR (95% CI)
T-stage (T1/T2 vs. T3/T4)	0.34	1.42 (0.69–2.42)
Histology stage (W/M vs. poorly)	0.16	3.61 (0.60–21.64)
Manual nuclear atypia grading (low vs. high)	0.23	2.55 (0.55–11.75)
Invasion depth (out vs. in)	0.51	0.56 (0.09–3.14)
Tumor diameter (< 5 cm vs. ≥ 5 cm)	0.62	0.37 (0.09–18.83)
NGAHIC (positive vs. negative)	*1.6 × 10* ^*−4*^	17.24 (3.93–75.60)

*CI* confidence interval, *HR* hazard ratio, *NGAHIC* image classifier, *out* out of serosa, in invasion of serosa, *W/M* well and moderate-differentiate

P value in italic is statistically significant, P < 0.05

## Discussion

Although INGA patients have a better prognosis than those with lymph node involved, INGA patients still suffered from disease recurrence, mostly seeding through peritoneal or hematogenous spread [24]. Once INGA patients experience recurrence, their lifespan is significantly decreased. The recurrent patients need more close attention, such as more aggressive treatment and advance care planning. Hence there is a need to identify patients with high-risk recurrence following surgery. Nuclear atypia refers to changes in nuclear morphological profiles, including nuclear appearance, size, or arrangement, and has proved to be useful hallmark of cancer prognosis and choice of adjuvant therapies determination, in different types of cancers clinicopathologically [6, 11–14, 19, 25]. However, human-based observations often suffered from inter- and intra-reader variation.

Computer-aid for automatic estimation by image analysis technology has been proved to mitigate the subjectivity by pathologists [6, 11, 12, 14, 25]. In this work, we exploited a computer-aid histomorphometric classifier for accurate prediction of recurrence of INGA patients. An image based models was constructed to extract features of nuclear shape, texture and orientation features from H&E stained TMA images. This designation could capture nuclear morphology features quantitatively and precisely in local tumor region. The data revealed that the more the heterogeneous nuclear features were related to the high risk for disease recurrence and worse prognosis of INGA patients. The Kaplan–Meier analysis along utilizing the log-rank test showed a strong association between the predictions of the image classifier and recurrence for D2. In addition, the tumor heterogeneity was also investigated across comparing the image classifier prediction ability on D2 and D3 (tumor punches from different parts of the same tumor). The image classifier was tent to be prognostic in both D2 (P = 1.6 × 10^−4^) and D3 (P = 0.02), respectively (Fig. 4h, i).

We also inspected the prognostic performance difference between the image classifier and the human-based nuclear atypia grade for INGA. However, the Kaplan–Meier analysis results with log-rank test showed no significant statistical association between the reader 1 human-based nuclear atypia grade estimation and survival outcome for D1 or D2 (P > 0.05), meanwhile a strong negative statistical relationship was observed with reader 2 and patient outcome for D2 (P = 0.004). In converse, A Kaplan–Meier analysis along utilizing the log-rank test showed a strong association between the predictions of the image classifier and recurrence for D2 (P < 0.05). Likewise, a multivariate Cox proportional survival analysis reported a HR of 17.24 (95% confidence interval: 3.93–75.60, P = 1.6 × 10^−4^). This could be illustrated by human estimation variability. Indeed, patients with early stage gastric adenocarcinoma exhibit a broad survival range, and the nuclear atypia stage only limited the survival outcome prediction, resulting in discordance diagnosis. Additionally, the morphological features for evaluating nuclear atypia grade are generally difficult to spot by human inspection, but can be identified by computer easily and effectively, such as shape/texture, nuclear arrangement et al. Furthermore, the nuclear atypia grading criteria and the prognostic values of nuclear atypia grade in INGA have not been defined clearly. Hence, each pathologist might be focus on different nuclear morphological profiles, such as nuclear shape (enlarged or hyperchromatic nuclei), nuclear area, disordered nuclear polarity, cytoplasmic mucin reduction or other features subjectively. Finally, different pathologists may have variable expertise evaluating or natural individual variation in their perception of colors, shapes, and relative nuclear pleomorphism/polarity proportions. Comparatively, the local nuclear features, encompassing local nuclear orientation, nuclear shape, and nuclear arrangement, were measured and extracted objectively and thereby the image classifier revealed a strong association with tumor outcomes in early gastric adenocarcinoma. Moreover, a significant correlation between HER2 overexpression and NGAHIC-positive has been observed in INGA. 81.3% of NGAHIC-positive carcinomas were positive for HER2 staining vs. that of 3.6% NGAHIC-negative cancers (P < 0.001). Meanwhile, the IHC staining revealed that the NGAHIC-positive patients have higher positive rate (75.7%) vs. that of in NGAHIC-negative patients (2.4%) for Ki67, with P < 0.001.

In this study, the local nuclear orientation features, indicating the heterogeneity of nuclear polarity, were found to be persistently activated and overexpressed with poor tumor outcomes for distinguishing high-risk recurrence patients and low-risk recurrences patients. Namely, the higher expression of nuclear polarity in the cell cultures, the poorer disease outcomes. Intuitively, aggressive tumors tent to exhibit relatively lower degree of structure and organization as rapid disorganized cell regeneration, compared with less aggressive cancer. These findings present a similar pattern of results as previous works [13, 14, 25]. Additionally, we also inspected the relationship between nuclear shape/texture features, nuclear architecture features and disease prognosis. In the INGA group, the most discriminative features also covered the nuclear shape feature (SD of perimeter ration) and the nuclear architecture feature (disorder of perimeter). This showed that both local anatomical structures (shape and cell nuclei) and local architecture of the tumor cell nucleus (Delaunay triangulation of the nuclear, e.t.) are associated with survival outcomes. Nuclear atypia, referring to the alternations in nuclear structure, such as shape, architecture, orientation, tend to be captured by the computer-extracted features quantitatively and used for cancer grading. These findings consistent with the previous researches stance that the nuclear shape and architecture appears to predictive of patient survival [2, 5, 6, 8–11, 14, 18].

The main contributions of this paper were summarized as follow. (1) In this study, it was a preliminary finding that the relationship between more aggressive clustered tumor computer-extracted H&E image features of nuclear cluster graph and recurrence were strong closed in INGA patients. To our best known, it has never been reported in the literature. (2) The simple binary histomorphometric image classifier could stratify the patient into different prognosis groups. Especially, the NGAHIC positive patients, identify high risk of recurrence patients by the image classifier, has worse disease outcome. This preliminary finding resulted in possibility for image classifier as prognosis marker to be used in potential clinic routine. We imagine with the help of NGAHIC, pathologist could identify high recurrent risk patients through H&E stained digital images, including biopsy or surgical specimen. Providing the accurate pathologic diagnosis, clinicians could make an individualized treatment, such as postoperative close chemotherapy and radiation therapy and follow-up. Certainly, NGAHIC needs to be tested in multicenter study of large samples.

We acknowledge the limitations of this work. We only utilized the 2 mm tissue microarrays, containing a relative small portion of tumor characteristics as composed to whole tumors, for digital assessment. However, recent scholars proposed that the important cell morphological diversity present in one tumor tissue could be obtained in tissue microarrays [26–28]. Furthermore, we will expand our study to whole-slide histopathology images as they contain large amount information and multi-view of tumor. Additionally, the entire cohort in our study is relative small and some of clinical parameters, such as nodal extracapsular extension, margin, were not included in for multivariate analysis. Future efforts will be made to investigate our model on multi-institutional study with considerable samples of INGA.

## Conclusion

In summary, we demonstrated that the histopathology image classifier based off local nuclear features, related to disorder of nuclear shape, arrangement, and orientation within the tumor cluster area, can predict recurrence and survival outcomes of INGA patients successfully. This capability is superior to the current practice utilization by nuclear atypia grade assessment by pathologists subjectively. Furthermore, our model could facilitate prognostic prediction based off the collected H&E stained slides routinely, and thereby contributing to the precision oncology, personalized cancer management and advance care planning. Future works will involve research on the response to treatment by analyzing the pathological images digitally.

## Additional files

**Additional file 1: Table S1.** All quantitative features list.

**Additional file 2: Table S2.** Summary of representative features by 3 different feature selection methods.

**Additional file 3: Table S3.** Comparative analysis of the image classifier and immunohistochemistry.
