# Supplementary material for: Nuclear shape, architecture and orientation features from H&E images are able to predict recurrence in node-negative gastric adenocarcinoma
Source: J Transl Med. 2019 Mar 18;17:92. doi: 10.1186/s12967-019-1839-x (PMC6423755; doi:10.1186/s12967-019-1839-x)
Supplement: Supplementary file 1 — Additional file 1: Table S1. All quantitative features list. [file 12967_2019_1839_MOESM1_ESM.docx]

Table S1 all quantitative features list

| # | **Feature Name** |
| --- | --- |
| 1 | Shape: Mean Area Ratio |
| 2 | Shape: Mean Distance Ratio |
| 3 | Shape: Mean Standard Deviation of Distance |
| 4 | Shape: Mean Variance of Distance |
| 5 | Shape: Mean Long/Short Distance Ratio |
| 6 | Shape: Mean Perimeter Ratio |
| 7 | Shape: Mean Smoothness |
| 8 | Shape: Mean Invariant Moment 1 |
| 9 | Shape: Mean Invariant Moment 2 |
| 10 | Shape: Mean Invariant Moment 3 |
| 11 | Shape: Mean Invariant Moment 4 |
| 12 | Shape: Mean Invariant Moment 5 |
| 13 | Shape: Mean Invariant Moment 6 |
| 14 | Shape: Mean Invariant Moment 7 |
| 15 | Shape: Mean Fractal Dimension |
| 16 | Shape: Mean Fourier Descriptor 1 |
| 17 | Shape: Mean Fourier Descriptor 2 |
| 18 | Shape: Mean Fourier Descriptor 3 |
| 19 | Shape: Mean Fourier Descriptor 4 |
| 20 | Shape: Mean Fourier Descriptor 5 |
| 21 | Shape: Mean Fourier Descriptor 6 |
| 22 | Shape: Mean Fourier Descriptor 7 |
| 23 | Shape: Mean Fourier Descriptor 8 |
| 24 | Shape: Mean Fourier Descriptor 9 |
| 25 | Shape: Mean Fourier Descriptor 10 |
| 26 | Shape: Standard Deviation Area Ratio |
| 27 | Shape: Standard Deviation Distance Ratio |
| 28 | Shape: Standard Deviation Standard Deviation of Distance |
| 29 | Shape: Standard Deviation Variance of Distance |
| 30 | Shape: Standard Deviation Long/Short Distance Ratio |
| 31 | Shape: Standard Deviation Perimeter Ratio |
| 32 | Shape: Standard Deviation Smoothness |
| 33 | Shape: Standard Deviation Invariant Moment 1 |
| 34 | Shape: Standard Deviation Invariant Moment 2 |
| 35 | Shape: Standard Deviation Invariant Moment 3 |
| 36 | Shape: Standard Deviation Invariant Moment 4 |
| 37 | Shape: Standard Deviation Invariant Moment 5 |
| 38 | Shape: Standard Deviation Invariant Moment 6 |
| 39 | Shape: Standard Deviation Invariant Moment 7 |
| 40 | Shape: Standard Deviation Fractal Dimension |
| 41 | Shape: Standard Deviation Fourier Descriptor 1 |
| 42 | Shape: Standard Deviation Fourier Descriptor 2 |
| 43 | Shape: Standard Deviation Fourier Descriptor 3 |
| 44 | Shape: Standard Deviation Fourier Descriptor 4 |
| 45 | Shape: Standard Deviation Fourier Descriptor 5 |
| 46 | Shape: Standard Deviation Fourier Descriptor 6 |
| 47 | Shape: Standard Deviation Fourier Descriptor 7 |
| 48 | Shape: Standard Deviation Fourier Descriptor 8 |
| 49 | Shape: Standard Deviation Fourier Descriptor 9 |
| 50 | Shape: Standard Deviation Fourier Descriptor 10 |
| 51 | Shape: Median Area Ratio |
| 52 | Shape: Median Distance Ratio |
| 53 | Shape: Median Standard Deviation of Distance |
| 54 | Shape: Median Variance of Distance |
| 55 | Shape: Median Long/Short Distance Ratio |
| 56 | Shape: Median Perimeter Ratio |
| 57 | Shape: Median Smoothness |
| 58 | Shape: Median Invariant Moment 1 |
| 59 | Shape: Median Invariant Moment 2 |
| 60 | Shape: Median Invariant Moment 3 |
| 61 | Shape: Median Invariant Moment 4 |
| 62 | Shape: Median Invariant Moment 5 |
| 63 | Shape: Median Invariant Moment 6 |
| 64 | Shape: Median Invariant Moment 7 |
| 65 | Shape: Median Fractal Dimension |
| 66 | Shape: Median Fourier Descriptor 1 |
| 67 | Shape: Median Fourier Descriptor 2 |
| 68 | Shape: Median Fourier Descriptor 3 |
| 69 | Shape: Median Fourier Descriptor 4 |
| 70 | Shape: Median Fourier Descriptor 5 |
| 71 | Shape: Median Fourier Descriptor 6 |
| 72 | Shape: Median Fourier Descriptor 7 |
| 73 | Shape: Median Fourier Descriptor 8 |
| 74 | Shape: Median Fourier Descriptor 9 |
| 75 | Shape: Median Fourier Descriptor 10 |
| 76 | Shape: Min / Max Area Ratio |
| 77 | Shape: Min / Max Distance Ratio |
| 78 | Shape: Min / Max Standard Deviation of Distance |
| 79 | Shape: Min / Max Variance of Distance |
| 80 | Shape: Min / Max Long/Short Distance Ratio |
| 81 | Shape: Min / Max Perimeter Ratio |
| 82 | Shape: Min / Max Smoothness |
| 83 | Shape: Min / Max Invariant Moment 1 |
| 84 | Shape: Min / Max Invariant Moment 2 |
| 85 | Shape: Min / Max Invariant Moment 3 |
| 86 | Shape: Min / Max Invariant Moment 4 |
| 87 | Shape: Min / Max Invariant Moment 5 |
| 88 | Shape: Min / Max Invariant Moment 6 |
| 89 | Shape: Min / Max Invariant Moment 7 |
| 90 | Shape: Min / Max Fractal Dimension |
| 91 | Shape: Min / Max Fourier Descriptor 1 |
| 92 | Shape: Min / Max Fourier Descriptor 2 |
| 93 | Shape: Min / Max Fourier Descriptor 3 |
| 94 | Shape: Min / Max Fourier Descriptor 4 |
| 95 | Shape: Min / Max Fourier Descriptor 5 |
| 96 | Shape: Min / Max Fourier Descriptor 6 |
| 97 | Shape: Min / Max Fourier Descriptor 7 |
| 98 | Shape: Min / Max Fourier Descriptor 8 |
| 99 | Shape: Min / Max Fourier Descriptor 9 |
| 100 | Shape: Min / Max Fourier Descriptor 10 |
| 101 | Texture: Mean contrast of R channel |
| 102 | Texture: SD contrast of R channel |
| 103 | Texture: Mean energy of R channel |
| 104 | Texture: SD energy of R channel |
| 105 | Texture: Mean entropy of R channel |
| 106 | Texture: SD entropy of R channel |
| 107 | Texture: Mean inverse variance of R channel |
| 108 | Texture: SD inverse variance of R channel |
| 109 | Texture: Mean invariant moment of R channel |
| 110 | Texture: SD invariant moment of R channel |
| 111 | Texture: Mean contrast of G channel |
| 112 | Texture: SD contrast of G channel |
| 113 | Texture: Mean energy of G channel |
| 114 | Texture: SD energy of G channel |
| 115 | Texture: Mean entropy of G channel |
| 116 | Texture: SD entropy of G channel |
| 117 | Texture: Mean inverse variance of G channel |
| 118 | Texture: SD inverse variance of G channel |
| 119 | Texture: Mean invariant moment of G channel |
| 120 | Texture: SD invariant moment of G channel |
| 121 | Texture: Mean contrast of B channel |
| 122 | Texture: SD contrast of B channel |
| 123 | Texture: Mean energy of B channel |
| 124 | Texture: SD energy of B channel |
| 125 | Texture: Mean entropy of B channel |
| 126 | Texture: SD entropy of B channel |
| 127 | Texture: Mean inverse variance of B channel |
| 128 | Texture: SD inverse variance of B channel |
| 129 | Texture: Mean invariant moment of B channel |
| 130 | Texture: SD invariant moment of B channel |
| 131 | Orientation: Mean contrast energy |
| 132 | Orientation: SD contrast energy |
| 133 | Orientation: Rang of contrast energy |
| 134 | Orientation: Mean contrast inverse moment |
| 135 | Orientation: SD contrast inverse moment |
| 136 | Orientation: Rang of contrast inverse moment |
| 137 | Orientation: Mean contrast average |
| 138 | Orientation: SD contrast average |
| 139 | Orientation: Rang of contrast average |
| 140 | Orientation: Mean contrast variance |
| 141 | Orientation: SD contrast variance |
| 142 | Orientation: Rang of contrast variance |
| 143 | Orientation: Mean contrast entropy |
| 144 | Orientation: SD contrast entropy |
| 145 | Orientation: Rang of contrast entropy |
| 146 | Orientation: Mean intensity average |
| 147 | Orientation: SD intensity average |
| 148 | Orientation: Rang of intensity average |
| 149 | Orientation: Mean intensity variance |
| 150 | Orientation: SD intensity variance |
| 151 | Orientation: Rang of intensity variance |
| 152 | Orientation: Mean intensity entropy |
| 153 | Orientation: SD intensity entropy |
| 154 | Orientation: Rang of intensity entropy |
| 155 | Orientation: Mean entropy |
| 156 | Orientation: SD entropy |
| 157 | Orientation: Rang of entropy |
| 158 | Orientation: Mean energy |
| 159 | Orientation: SD energy |
| 160 | Orientation: Rang of energy |
| 161 | Orientation: Mean correlation |
| 162 | Orientation: SD correlation |
| 163 | Orientation: Rang of correlation |
| 164 | Orientation: Mean information measure 1 |
| 165 | Orientation: SD information measure 1 |
| 166 | Orientation: Rang of information measure 1 |
| 167 | Orientation: Mean information measure 2 |
| 168 | Orientation: SD information measure 2 |
| 169 | Orientation: Rang of information measure 2 |
| 170 | Voronoi: Area Standard Deviation |
| 171 | Voronoi: Area Average |
| 172 | Voronoi: Area Minimum / Maximum |
| 173 | Voronoi: Area Disorder |
| 174 | Voronoi: Perimeter Standard Deviation |
| 175 | Voronoi: Perimeter Average |
| 176 | Voronoi: Perimeter Minimum / Maximum |
| 177 | Voronoi: Perimeter Disorder |
| 178 | Voronoi: Chord Standard Deviation |
| 179 | Voronoi: Chord Average |
| 180 | Voronoi: Chord Minimum / Maximum |
| 181 | Voronoi: Chord Disorder |
| 182 | Delaunay: Side Length Minimum / Maximum |
| 183 | Delaunay: Side Length Standard Deviation |
| 184 | Delaunay: Side Length Average |
| 185 | Delaunay: Side Length Disorder |
| 186 | Delaunay: Triangle Area Minimum / Maximum |
| 187 | Delaunay: Triangle Area Standard Deviation |
| 188 | Delaunay: Triangle Area Average |
| 189 | Delaunay: Triangle Area Disorder |
|  | NOTE: SD, Standard Deviation |
