# Supplementary material for: Nuclear shape, architecture and orientation features from H&E images are able to predict recurrence in node-negative gastric adenocarcinoma
Source: J Transl Med. 2019 Mar 18;17:92. doi: 10.1186/s12967-019-1839-x (PMC6423755; doi:10.1186/s12967-019-1839-x)
Supplement: Supplementary file 2 — Additional file 2: Table S2. Summary of representative features by 3 different feature selection methods. [file 12967_2019_1839_MOESM2_ESM.docx]

Table S2 Summary of representative features by 3 different feature selection methods.

| Feature bin type | Descriptors |
| --- | --- |
| MRMR |  |
| Nuclear Orientation Map | SD of energy |
| Nuclear Orientation Map | range of contrast entropy |
| Nuclear shape | SD of smoothness |
| Nuclear texture | mean of invariant moment R channel |
| Nuclear texture | SD of inverse variance R channel |
| RF |  |
| Nuclear shape | SD of Fourier perimeter ratio |
| Nuclear Orientation Map | range of contrast energy |
| Nuclear texture | SD of invariant moment |
| Nuclear Orientation Map | mean of contrast entropy |
| Voronoi Diagram | Min/Max perimeter |
| WRST |  |
| Nuclear Orientation Map | range of intensity entropy |
| Nuclear Orientation Map | range of intensity energy |
| Nuclear shape | SD of perimeter ration |
| Nuclear Orientation Map | SD of intensify average |
| Delaunay Triangulation | disorder of perimeter |

SD: Standard Deviation, MRMR: Minimum redundancy maximum relevance, RF: Random Forest, WRST: Wilcoxon rank sum test
