# Supplementary material for: Nuclear shape, architecture and orientation features from H&E images are able to predict recurrence in node-negative gastric adenocarcinoma
Source: J Transl Med. 2019 Mar 18;17:92. doi: 10.1186/s12967-019-1839-x (PMC6423755; doi:10.1186/s12967-019-1839-x)
Supplement: Supplementary file 3 — Additional file 3: Table S3. Comparative analysis of the image classifier and immunohistochemistry. [file 12967_2019_1839_MOESM3_ESM.docx]

**Table S3. Comparative analysis of the image classifier and immunohistochemistry**

|  | **Image classifier** | | | |  |
| --- | --- | --- | --- | --- | --- |
| **variable** | **Positive(n=16)** | | **Negative(n=84)** | | **P*** |
| HER2 positive | 13 | (81.3%) | 3 | (3.6%) | <0.001 |
| negative | 3 | (18.7%) | 81 | (96.4%) |  |
| Ki67 positive | 12 | (75.0%) | 2 | (2.4%) | <0.001 |
| negative | 4 | (25.0%) | 82 | (97.6%) |  |

*: chi-square test.
